# Identification of key methylation differentially expressed genes in posterior fossa ependymoma based on epigenomic and transcriptome analysis

**DOI:** 10.1186/s12967-021-02834-1

**Published:** 2021-04-26

**Authors:** Guanyi Wang, Yibin Jia, Yuqin Ye, Enming Kang, Huijun Chen, Jiayou Wang, Xiaosheng He

**Affiliations:** 1 Department of Neurosurgery, Xijing Hospital, Airforce Military Medical University (Fourth Military Medical University), Xi’an, 710032 China; 2 Department of Neurosurgery, PLA 163Rd Hospital (Second Affiliated Hospital of Hunan Normal University), Changsha, 410000 China

**Keywords:** Posterior fossa ependymoma, Epigenome, Transcriptome, Differential genes, WGCNA

## Abstract

**Background:**

Posterior fossa ependymoma (EPN-PF) can be classified into Group A posterior fossa ependymoma (EPN-PFA) and Group B posterior fossa ependymoma (EPN-PFB) according to DNA CpG island methylation profile status and gene expression. EPN-PFA usually occurs in children younger than 5 years and has a poor prognosis.

**Methods:**

Using epigenome and transcriptome microarray data, a multi-component weighted gene co-expression network analysis (WGCNA) was used to systematically identify the hub genes of EPN-PF. We downloaded two microarray datasets (GSE66354 and GSE114523) from the Gene Expression Omnibus (GEO) database. The Limma R package was used to identify differentially expressed genes (DEGs), and ChAMP R was used to analyze the differential methylation genes (DMGs) between EPN-PFA and EPN-PFB. GO and KEGG enrichment analyses were performed using the Metascape database.

**Results:**

GO analysis showed that enriched genes were significantly enriched in the extracellular matrix organization, adaptive immune response, membrane raft, focal adhesion, NF-kappa B pathway, and axon guidance, as suggested by KEGG analysis. Through WGCNA, we found that MEblue had a significant correlation with EPN-PF (R = 0.69, P = 1 × 10^–08^) and selected the 180 hub genes in the blue module. By comparing the DEGs, DMGs, and hub genes in the co-expression network, we identified five hypermethylated, lower expressed genes in EPN-PFA (ATP4B, CCDC151, DMKN, SCN4B, and TUBA4B), and three of them were confirmed by IHC.

**Conclusion:**

ssGSEA and GSVA analysis indicated that these five hub genes could lead to poor prognosis by inducing hypoxia, PI3K-Akt-mTOR, and TNFα-NFKB pathways. Further study of these dysmethylated hub genes in EPN-PF and the pathways they participate in may provides new ideas for EPN-PF treatment.

**Supplementary Information:**

The online version contains supplementary material available at 10.1186/s12967-021-02834-1.

## Introduction

Ependymoma (EPN) is a rare neuroepithelial tumor that occurs in both adults and children. As the third most common central nervous system tumor in children, it accounts for 10% of primary intracranial tumors in children, and two-thirds of the tumors are located in the posterior fossa (PF) [1, 2]. Based on histopathological criteria, the World Health Organization (WHO) classifies ependymomas as grades II and III tumors. However, whether this classification has guiding significance for prognosis has been controversial. Recently, according to the DNA CpG island methylation profile status and gene expression, posterior fossa ependymoma (EPN-PF) has been classified into two subtypes: Group A, posterior fossa ependymoma (EPN-PFA), and Group B, posterior fossa ependymoma (EPN-PFB) [3].

Compared to EPN-PFB, EPN-PFA exhibits CpGi hypermethylation, H3K27me3 reduction, global DNA hypomethylation, and EZHIP expression [4–7]. Clinical observations reveal that EPN-PFA usually occurs in children younger than 5 years and has a poor prognosis. Conversely, EPN-PFB usually occurs in children and adults older than 5 years, and the prognosis is relatively good [3, 8, 9]. At present, more in-depth studies are available about H3K27me3 and its mechanism of deletion. With the discovery of EZHIP, trials of various targeted drugs, such as the DNA methylation inhibitor decitabine [9, 10], selective EZH2 inhibitor GSK343, histone deacetylase inhibitor (HDACi) SAHA [11], have been initiated [4, 12].

To date, there are few studies about gene level targets. To the best of our knowledge, our study is the first to perform multi-omic weighted gene co-expression network analysis (WGCNA) to identify the hub genes of EPN-PF using both transcriptome- and epigenome-wide microarray data. The purpose of this study was to identify the differential hub genes between EPN-PFA and EPN-PFB and to explore their influence on prognosis from immunity and the pathways they participate.

## Methods

### Datasets

**Fig. 1:**
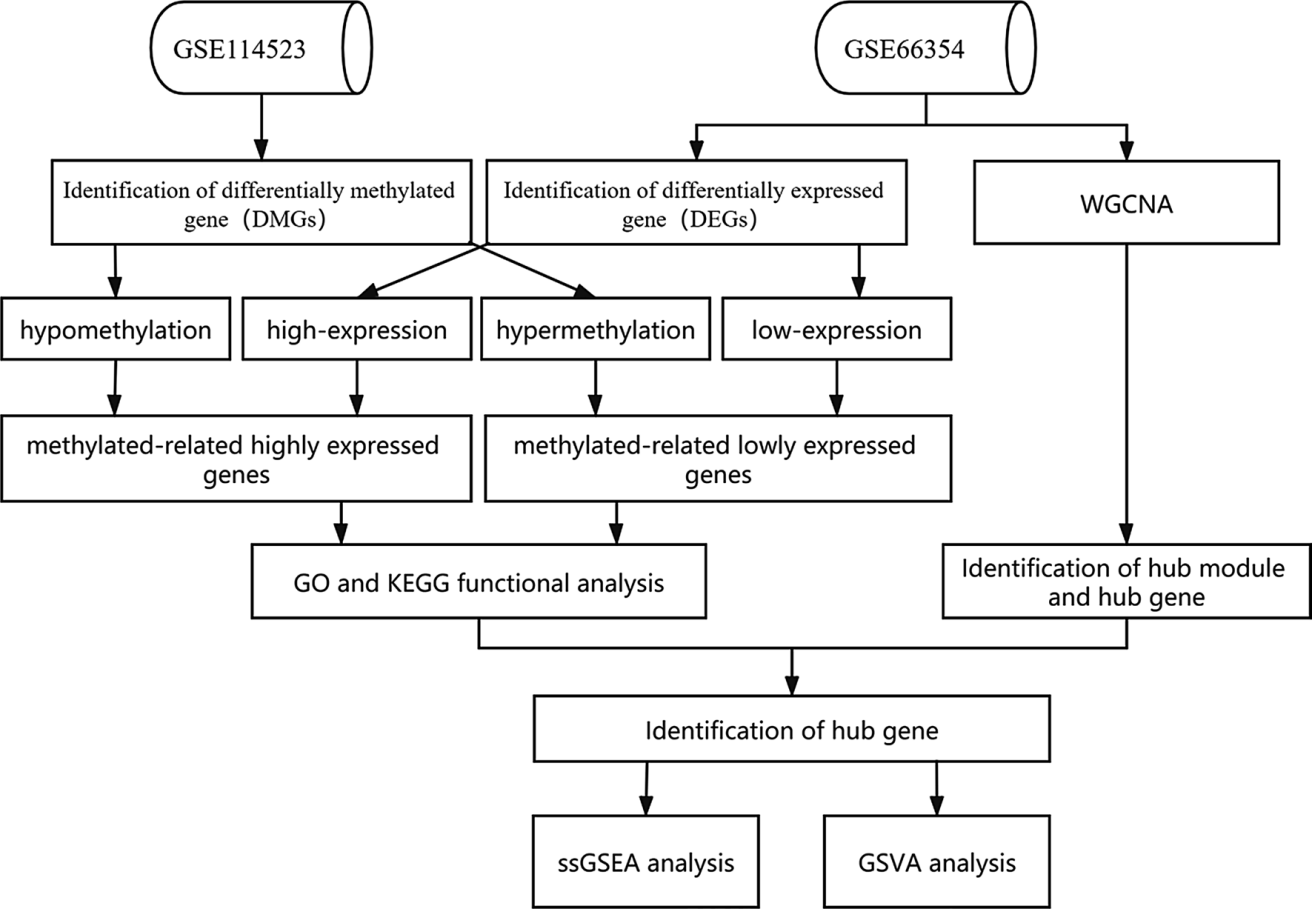
Study workflow

We downloaded two microarray datasets from the Gene Expression Omnibus (GEO) database. The dataset (GSE66354) [13] included gene expression profiles from surgical tumor and normal brain samples (n = 149) using Affymetrix HG-U133plus2 chips (Platform GPL570). We obtained the gene expression profiles of EPN-PFA (n = 29) and EPN-PFB (n = 26) from the dataset. In addition, we obtained DNA methylation profiles from 43 EPN-PFA and 12 EPN-PFB patients from the data set (GSE114523) [14]. The workflow of this study is shown in Fig. 1.

### Combined analysis of DNA methylation and transcriptome

DNA methylation causes transcriptional silencing to regulate gene expression. EPN-PF was also divided into two subtypes according to the level of CpG island methylation, and the prognosis was significantly different [15]. Therefore, we used GSE66354 and GSE114523 to find the hub-methylated genes.

The Limma R package was used to identify differentially expressed genes (DEGs), and the differential expression threshold was |logFC|> 1 & P < 0.05. We used the ChAMP R package to analyze the differential methylation genes (DMGs). The screening conditions were as follows: the β value of one group should be less than 0.2, whereas the other group should be greater than 0.3 and adjusted to P < 0.05. Next, we identified the EPN-PF-related DMGs, including methylated-related highly expressed genes and methylated-related lowly expressed genes.

Lastly, the Metascape database (www.metascape.org) [16] was used for functional and pathway enrichment analyses, Gene Ontology (GO) analysis, and Kyoto Genome Encyclopedia (KEGG) pathway analysis. Protein–protein interaction (PPI) network was carried out for the genes we obtained above. A Min overlap ≥ 3 & P ≤ 0.01 was considered statistically significant.

### Weighted gene co-expression network construction

We used the raw microarray data from GSE66354 to construct the gene co-expression networks using the WGCNA package [17]. By constructing a weighted gene co-expression network, we can find the gene modules of co-expression, and explore the relationship between gene network and phenotype as well as the core genes in the network. A matrix of similarity was constructed by calculating the correlations of all pairs of genes, then we ranked them by variance size and screened the top 5000 genes for the next analysis. The missing values were filled using the Impute package. The correlation between genes was calculated by the Pearson correlation matrix and the means of the connecting rod. An appropriate soft-thresholding power β was selected by using the integrated function (pickSoftThreshold) in the WGCNA package; here, the soft thresholding power is 9. The weighted adjacency matrix was transformed into a topological overlap degree matrix (TOM) to estimate the network connectivity, and the hierarchical clustering method was used to construct the clustering tree structure of the TOM matrix [18]. Different branches of the clustering tree represent different gene modules, and different colors represent different modules. Based on the weighted correlation coefficient of genes, genes are classified according to expression patterns, and genes with similar patterns are grouped into one module, whereas tens of thousands of genes are grouped into multiple modules through gene expression patterns [19].

### Identification of hub modules and hub genes

To identify the hub modules, we performed principal component analysis for module eigengenes (ME) and evaluated the Pearson correlation analysis between MEs and phenotype of EPN-PF. The most significant correlation modules were identified as hub modules. Subsequently, the genes within such modules were matched with EPN-PF-related DMGs to identify EPN-PF-related hub DMGs.

### Subjects and specimen collection

Between July 2017 and June 2020, we obtained six pairs of EPN-PF tissues from 12 patients at the Department of Neurosurgery, Xijing Hospital, Air Force Military Medical University. After surgery, the tumor tissue was preserved at 80 °C until use. This study was approved by the ethics committee of Xijing Hospital and the number is KY20202060-F-1.

### Immunohistochemistry

To determine the pathological subtype of each tumor, tumor sections were processed for immunostaining with antibodies against H3K27me3 (Rabbit, ThermoFisher, PA5-31817, 1:100). The cases with global reduction of H3K27me3 were considered EPN-PFA; in contrast, cases with H3K27me3 nuclear immunopositivity to be EPN-PFB. Then, in order to verify the expression level of the hub genes in two subtypes of EPN-PF, we immunostained the tumor sections with antibodies against ATP4B(Mouse, Invitrogen, MA3-923, 1:2000), CCDC151(Rabbit, ThermoFisher, PA5-62991, 1:1000), DMKN(Rabbit, Abcam, ab246965, 1:200), and SCN4B(Rabbit, Abcam, ab219816, 1:100).

The tumor tissue was immersed in 4% paraformaldehyde at 4 °C overnight, dehydrated with alcohol, and embedded in paraffin. Then, 5 μm thick sections were prepared using a microtome (Leica, Nussloch, Germany) and dried overnight at 94 °C. We selected 10 sections from each tissue and deparaffinized the sections using alcohol and dimethylbenzene. We then incubated the sections in citric acid antigen recovery buffer (pH = 6.0) at 95 °C for 10 min. Next, we incubated the sections in phosphate buffer saline (PBS)with 1% donkey serum albumin and 0.3% Triton X-100 at 25℃for 30 min. After preparation, the sections were dewaxed with xylene and rehydrated with a reduced alcohol solution. The sections were immersed in citrate buffer solution (pH 6.0), heated at high temperature in a microwave oven for 8 min, then taken out and cooled to room temperature and washed with PBS. Endogenous peroxide activity was quenched with 3% hydrogen peroxide for 15 min. Sheep serum (5%) was added to the slices and placed in a wet box at room temperature for 20 min and washed with PBS. The sections were first incubated with the primary antibodies mentioned above in PBS at 4 °C overnight. After washing, the tumor sections were treated with the corresponding secondary antibodies for 1 h at room temperature, and further incubated with HRP-labeled streptavidin. After washing, we added 50–100 μL of 3,3′-Diaminobenzidine(DAB) working solution to the sections. After color development, the sections were washed in distilled water, soaked in hematoxylin for 1–3 min, washed with distilled water, and then turned blue with PBS. Lastly, we dehydration, transparent, and covered the sections and then examined them under a microscope.

The sections were evaluated using the AxioVision Rel.4.6 computerized image analysis system assisted by an automatic measurement program (Nikon). The mean absorbance of the sections was determined as a measure of the strength of staining signals, as measured per positive pixel. The data were analyzed by t-test, all statistical analyses were conducted using GraphPad Prism 7 and *P* < 0.05 was considered statistically significant. Thus, we evaluated the expression of hub genes in different subtypes.

### Functional enrichment analysis

The single-sample gene set enrichment (ssGSEA) algorithm was performed based on the specific marker gene expression information of immune cells [20]; here, we used it to quantify the abundance of each tumor microenvironment (TME) cell infiltration. The adjusted enrichment scores calculated by ssGSEA analyses were used to represent the abundance of each TME infiltration cell. We evaluated 28 human TME cell subtypes, including B cells, CD8+ T cells, T helper cells, CCR, pDCs, and Th1 cells. The immune infiltration patterns and antigen-presenting activation levels were represented by the ssGSEA score. Then, the EPN-PF-related hub DMGs were analyzed via ssGSEA and gene set variation analysis (GSVA) [21] algorithm to comprehensively score each gene set to evaluate the potential biological function changes of different samples. The dataset we got from GSEA database Msigdb database. *P* < 0 0.05, gene size greater than or equal to 15 and enrichment score (ES) > 0.3 were set as the cut‐off value.

## Results

### Identification of EPN-PF-related DMGs

**Fig. 2:**
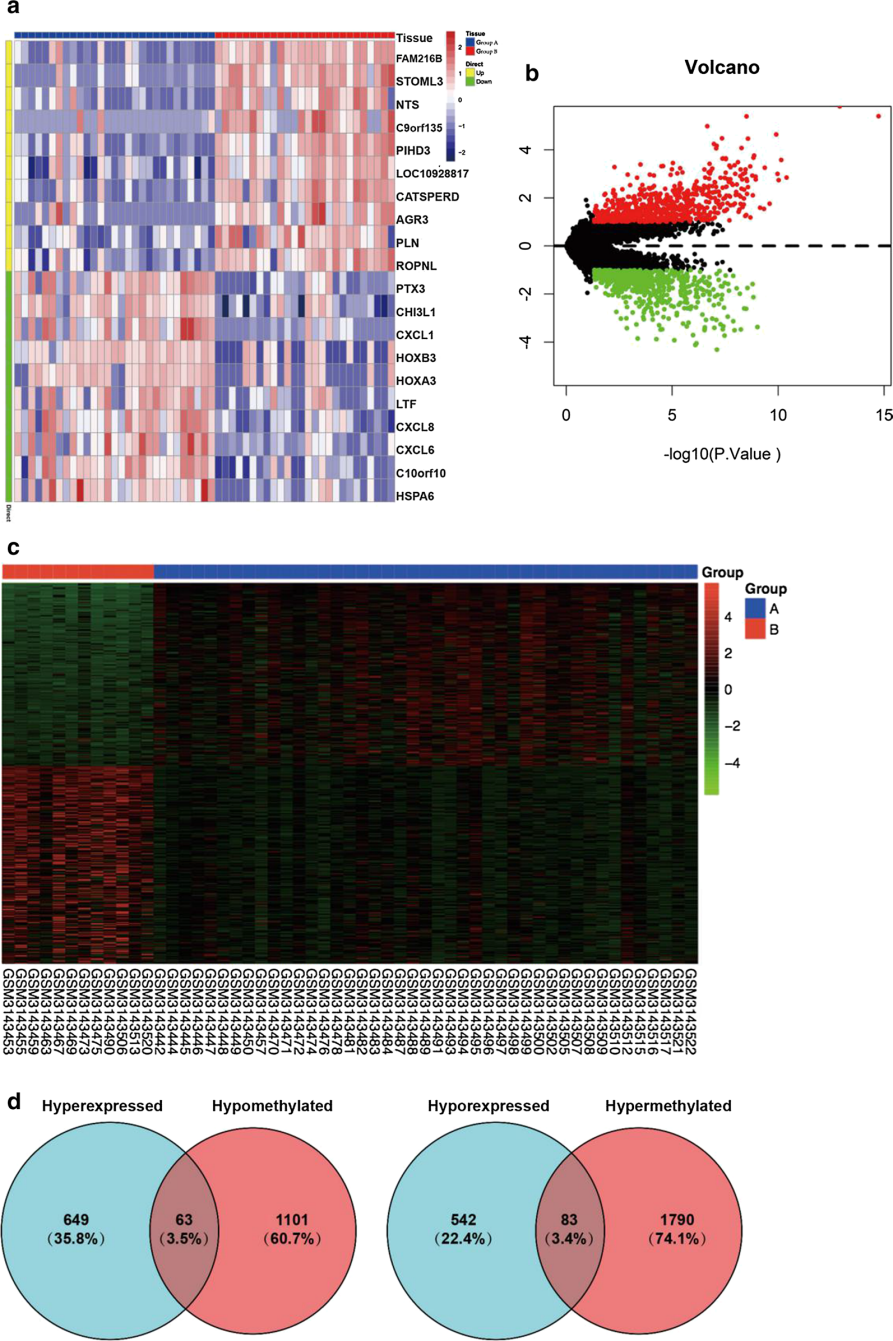
Dentification of EPN-PF-related DMGs. **a** The top 10 up- and down-regulated DEGs analyzed from the dataset of GSE66354. **b** Volcano plot for DEGs between two subtype of EPN-PF. **c** Heatmap of DMGs analyzed from the dataset of GSE114523, The β value of one group should be less than 0.2 and that of the other group should be greater than 0.3 and adjusted to P < 0.05. **d** The Venn diagram showed 63 methylated-related highly expressed genes and 83 methylated-related lowly expressed genes

By analyzing the GSE66354 dataset, we found that 712 genes were upregulated and 625 genes were downregulated in all 1337 DEGs between the two EPN-PF subtypes (Additional file 1: Table S1). The top 10 up- and down-regulated DEGs are provided in Fig. 2a and b. In the methylation profiling dataset of GSE114523, we found 1873 hypermethylated genes and 1164 hypomethylated genes (Fig. 2c and Additional file 2: Table S2). Then, using a Venn diagram, 63 methylated-related highly expressed genes (Additional file 3: Table S3) and 83 methylated-related lowly expressed genes(Additional file 4: Table S4) were revealed (Fig. 2d). Collectively, 146 aberrantly methylated DEGs were identified as EPN-PF-related DMGs.

### GO term, KEGG pathway, and PPI analysis

**Fig. 3:**
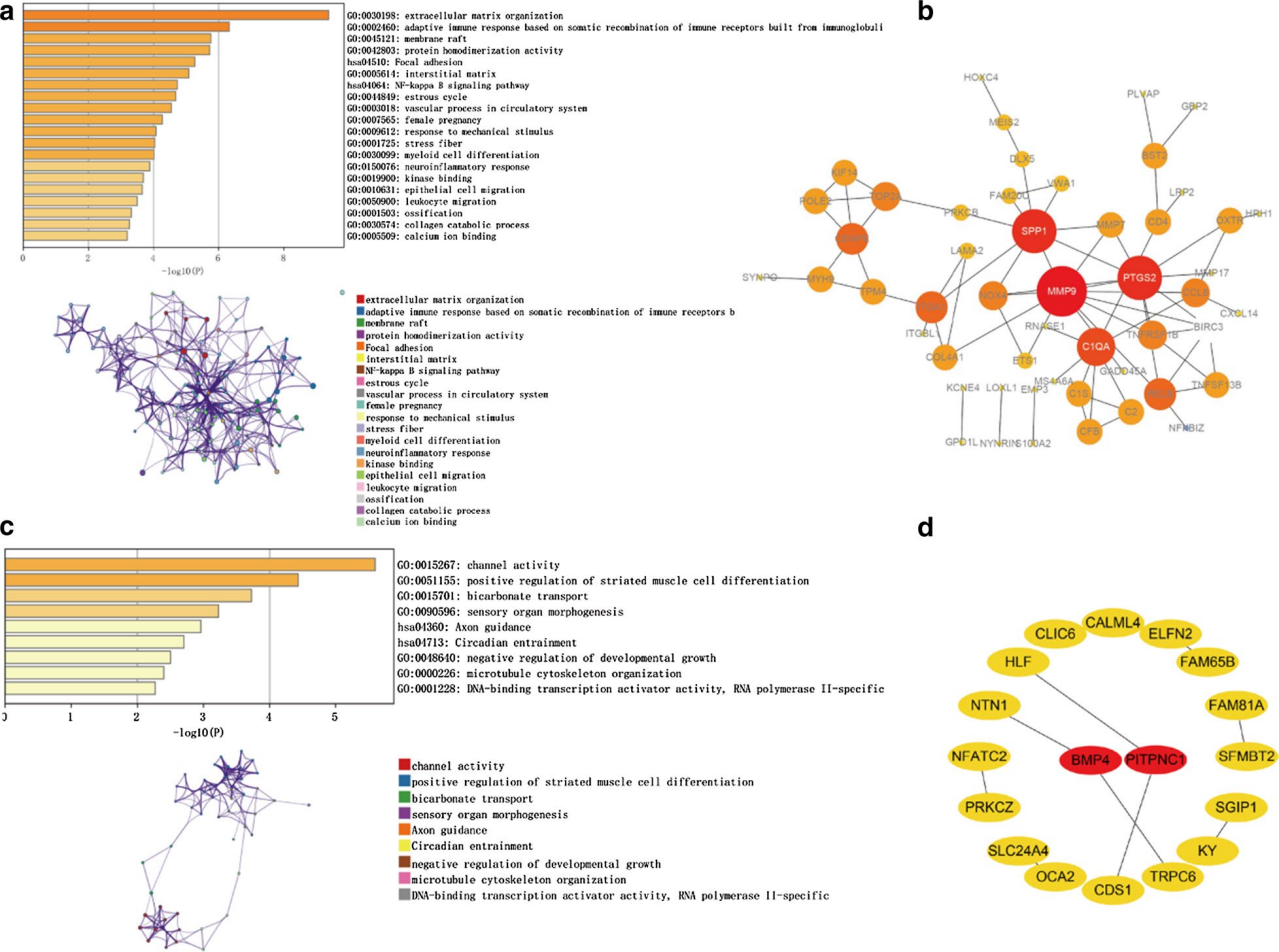
GO term, KEGG pathway, and PPI analysis. **a** The significant GO terms and KEGG pathway of the methylated-related lowly expressed genes. **b** The significant modules of methylated-related lowly expressed genes through PPI network analysis. Color represents connectivity. The higher the connectivity, the darker the color. **c** The significant GO terms and KEGG pathway of the methylated-related highly expressed genes. **d** The significant modules of methylated-related highly expressed genes through PPI network analysis. Color represents connectivity. The higher the connectivity, the darker the color

To further investigate the biological functions and signaling pathways involved in the occurrence and development of diseases, we analyzed both methylated-related highly expressed genes and methylated-related lowly expressed genes through GO, KEGG, and PPI analyses. The significant GO terms and KEGG pathway are shown in Fig. 3a and c. As shown in the GO analysis, the EPN-PF-related DMGs were remarkably enriched in extracellular matrix organization, adaptive immune response, and membrane raft. Furthermore, we found that the focal adhesion, NF-kappa B pathway, and axon guidance were significantly enriched as suggested by KEGG analysis. Through the PPI network analysis, we obtained two significant modules, which are shown in Fig. 3b and d.

### WGCNA and identification of hub modules

**Fig. 4:**
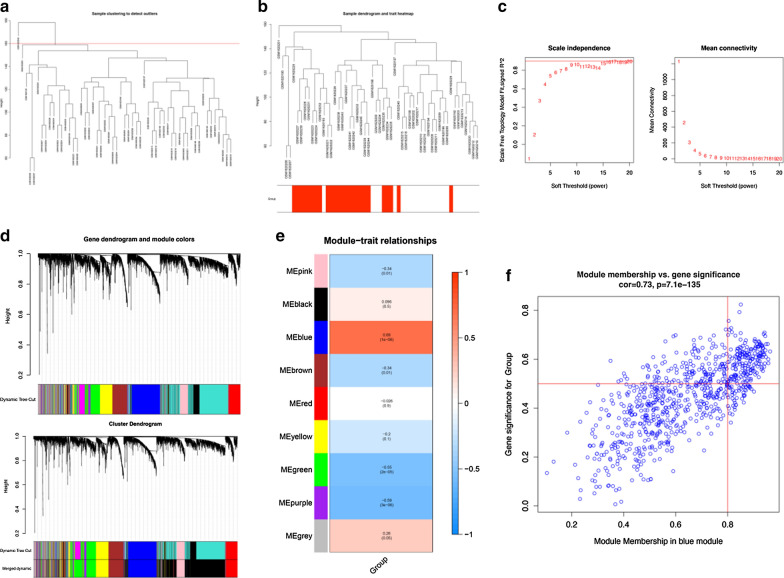
Weighted gene co-expression network analysis (WGCNA). **a** Clustering dendrogram of 55 samples. Sample clustering revealed that that sample GSM1620246 required removal from subsequent analysis due to outliers. **b**, **c** Soft-threshold power for WGCNA. **d** Dendrogram of all expressed genes in the top 25% of variance clustered based on a dissimilarity measure (1 − TOM). **e** Heatmap of correlation between ME and EPN-PF modules. **f** A scatterplot of Gene Significance (GS) for group vs. Module Membership (MM) in the blue module. There is a highly significant correlation between GS and MM in this module. 180 hub genes were selected in the blue module

As shown in Fig. 4a, sample clustering revealed that sample GSM1620246 required removal from subsequent analysis due to outliers. Subsequently, we included the remaining 54 samples in the WGCNA analysis and identified nine modules on the thresholding power of β = 9 (Fig. 4b–d). Among these, we identified the blue module, consisting of 805 genes, as highly correlated with the EPN localization (R = 0.69, p = 1E−08, Fig. 4e). Via Fig. 4f, we can find the correlation between the blue module and gene expression profiles on abscissa and the correlation between the gene and the tumor group on Ordinate. Combining the two coordinate, we confirmed there is a highly significant correlation between GS and MM in the blue module and then selected the 180 hub genes in the blue module (Additional file 5: Table S5).

### Identification of EPN-PF-related hub DMGs

**Fig. 5:**
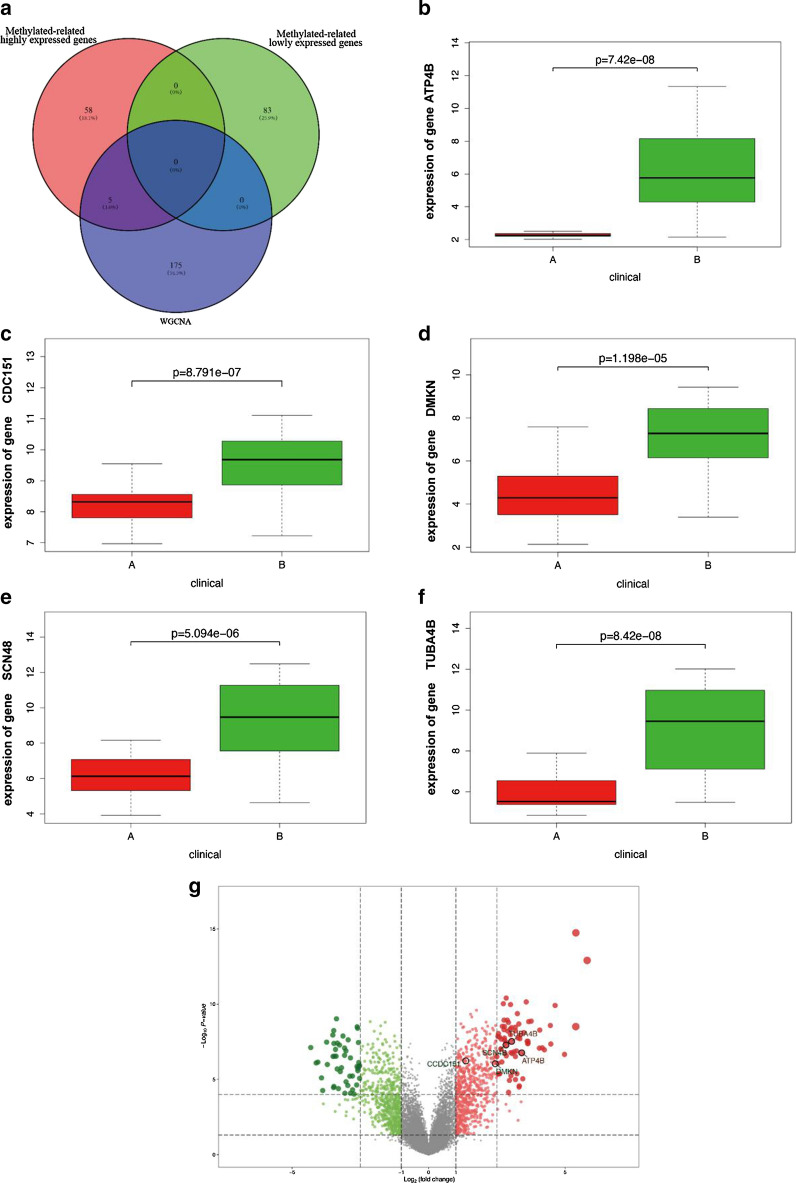
Identification of EPN-PF-related hub DMGs. **a** The Venn diagram shows 5 methylated-related highly expressed genes by comparing the EPN-PF-related DMGs and the hub genes in WGCNA that were identified as EPN-PF-related hub DMGs. **b**–**f** The relative expression levels of ATP4B (*P* = 7.42E-08), CCDC151 (*P* = 8.791E−07), DMKN (*P* = 1.198E−05), SCN4B (*P* = 5.904E−06), and TUBA4B (*P* = 8.42E−08) were significantly decreased in EPN-PFA cases compared with EPN-PFB cases. **g** The volcano plot for EPN-PF related hub DMGs between two subtypes of EPN-PF

Through the screening above, we compared the EPN-PF-related DMGs and the blue hub-related genes we obtained in WGCNA, and identified five genes as EPN-PF-related hub DMGs: ATP4B, CCDC151, DMKN, SCN4B, and TUBA4B (Fig. 5a–g).

### Verification of EPN-PF-related hub DMGs expression by IHC

**Fig. 6:**
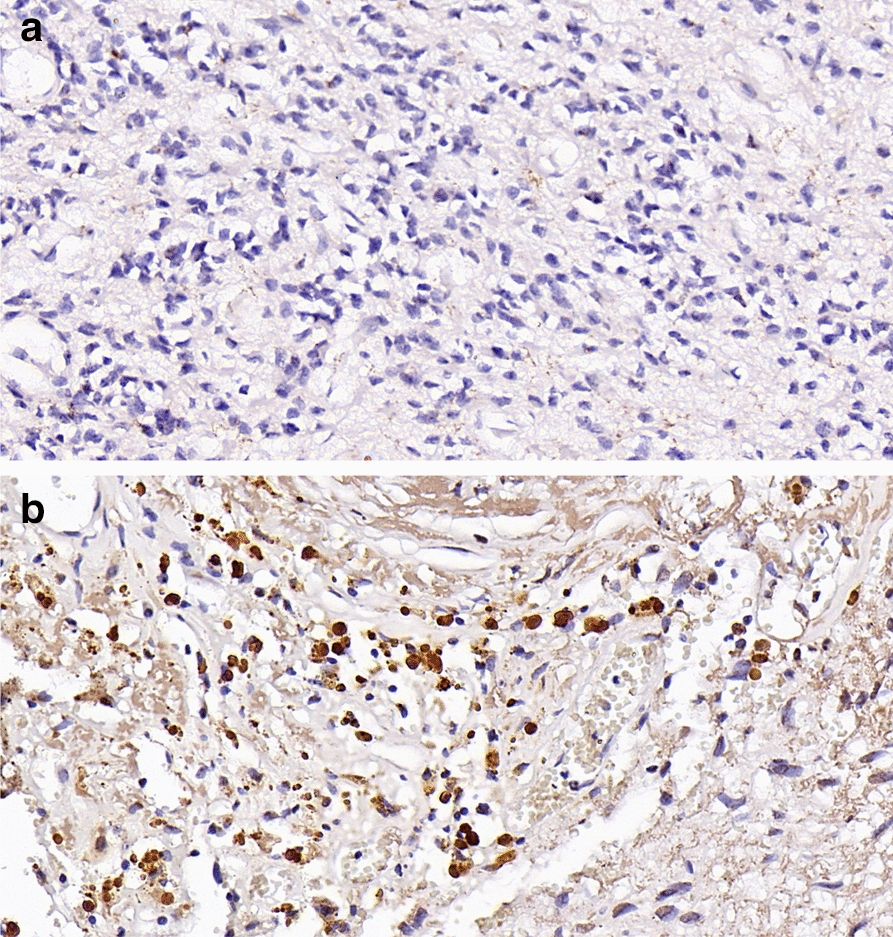
Identification of the two subtypes of EPN-PF. A, EPN-PFA: H3K27me3 nuclear staining is lost in the tumor cells. B, EPN-PFB: H3K27me3 nuclear staining is intact in the tumor cells

**Fig. 7:**
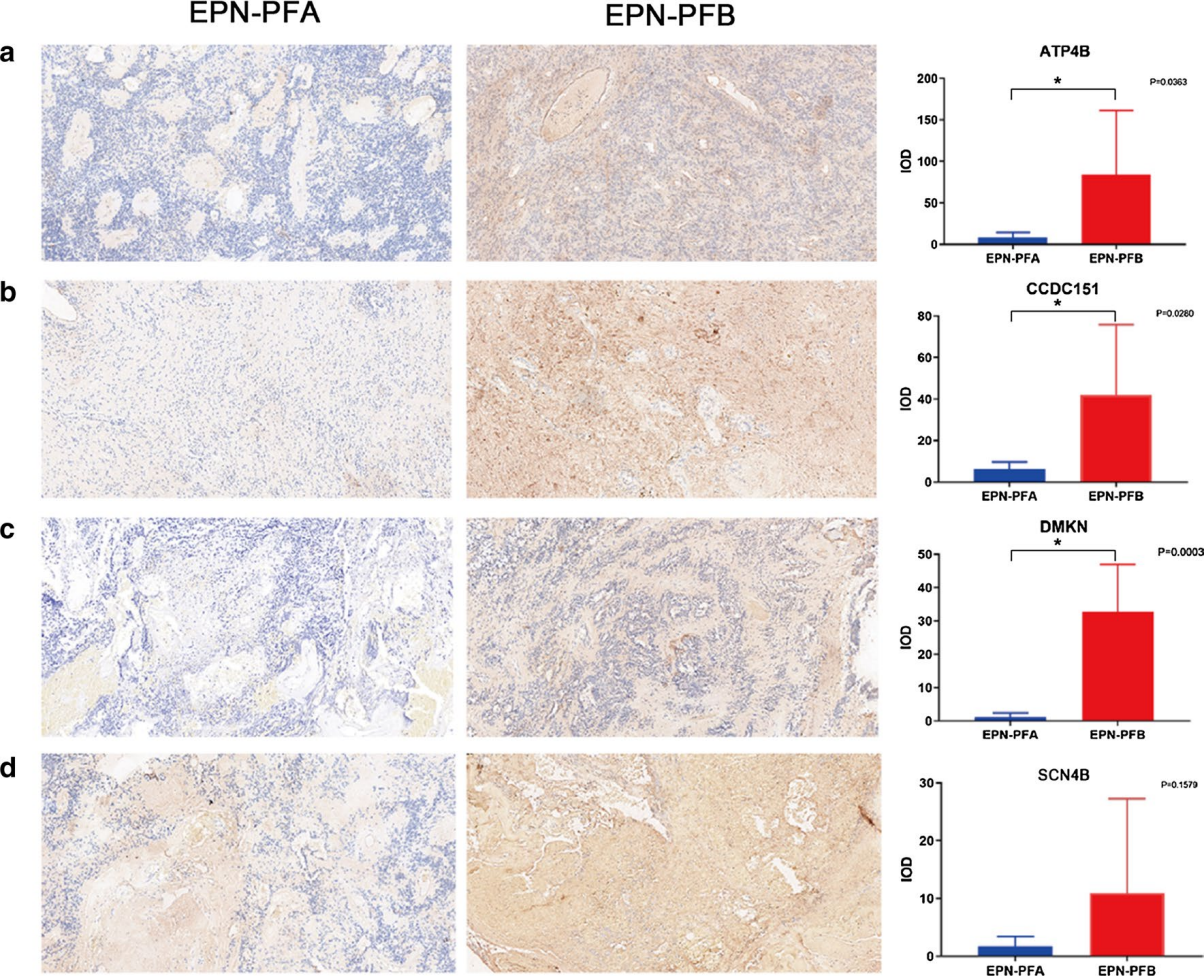
Comparison of gene expression between two subtypes of EPN-PF. **a** Representative photomicrographs stained with ATP4B. The expression of ATP4B in EPN-PFB was significantly higher than that in EPN-PFA (*P* = 0.0363). **b** Representative photomicrographs stained with CCDC151. The expression of CCDC151 in EPN-PFB was significantly higher than that in EPN-PFA (*P* = 0.0280). **c** Representative photomicrographs stained with DMKN. The expression of DMKN in EPN-PFB was significantly higher than that in EPN-PFA (*P* = 0.0003). **d** Representative photomicrographs stained with SCN4B.The expression of SCN4B in EPN-PFB was higher than that in EPN-PF, but the difference was no statistically significant (*P* = 0.0363)

Using an IHC assay to detect the expression levels of the hub DMGs obtained above, we verified the reliability of the bioinformatics analysis results. First, we identified the subtypes of the tumors we obtained (Fig. 6). As shown in Fig. 7, ATP4B, CCDC151, DMKN, and SCN4B levels were decreased in EPN-PFA compared with EPN-PFB, which was consistent with the predictions. Except for SCN4B (*P* = 0.1579), the differences in the other three were statistically significant (*P* < 0.05).

### Functional enrichment analysis

**Fig. 8:**
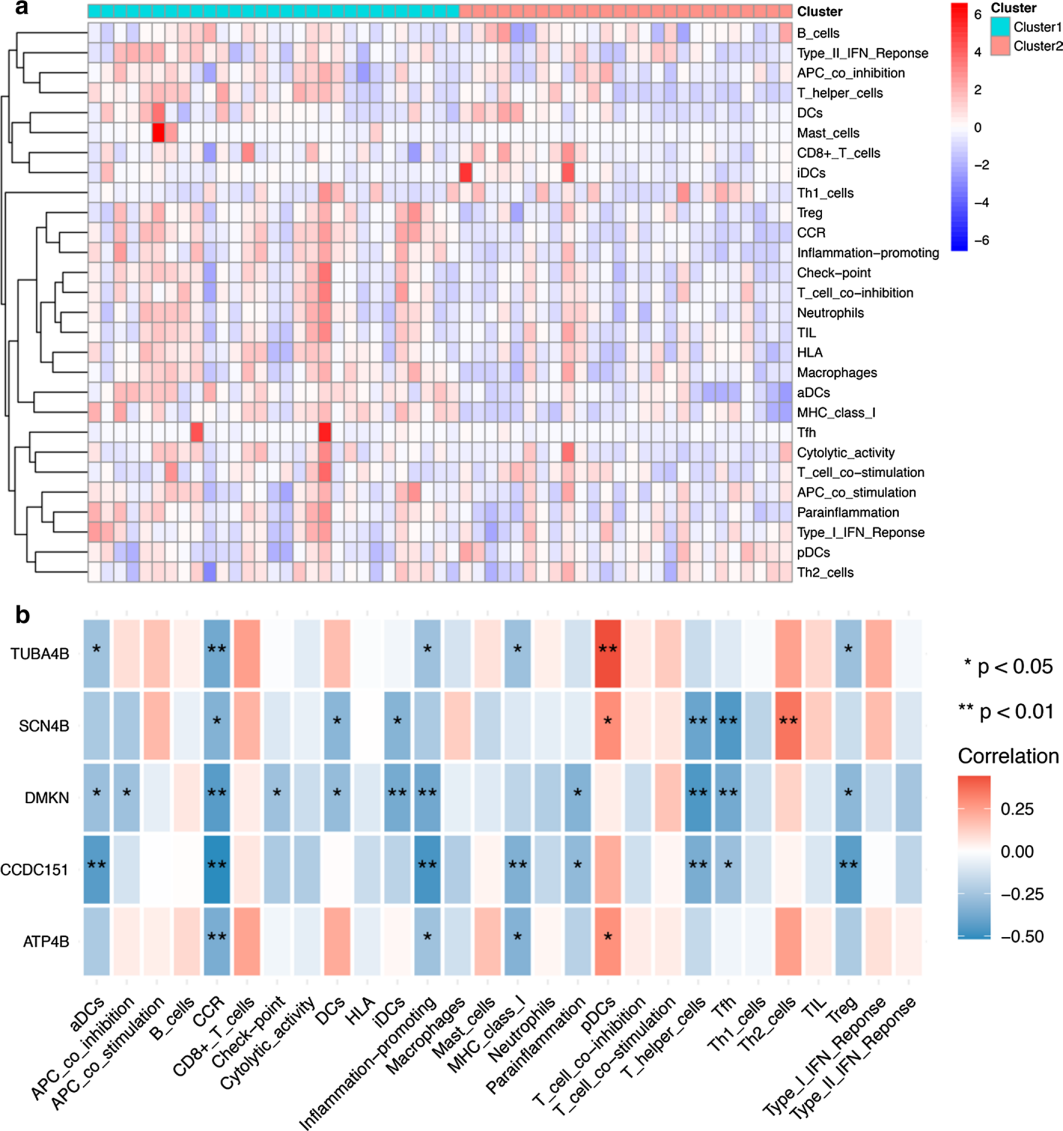
Functional enrichment analysis. **a** Landscape evaluation of 28 TME cell infiltration in two subtypes of EPN-PF samples. **b** The relationship between the 5 EPN-PF related hub DMGs and each TME infiltration cell type. Red, positive; Blue, negative

To explore the roles of key molecules identified in the TME immune cell infiltration, we evaluated the landscape of 28 TME cell infiltration in two subtypes of EPN-PF samples. As shown in Fig. 8a, there were remarkably differences in immune cells between the two subtypes. To further explore the relationship between the five EPN-PF-related hub DMGs and TME infiltrating cells, we correlated them with TME infiltrating cells. Spearman correlation analyses revealed a significantly correlation between these genes and TME infiltrating cells (Fig. 8b and Additional file 6; Table S6). In Fig. 8b, we could see directly that TUBA4B exhibited a prominent positive correlation with Plasmacytoid dendritic cells(pDCs), SCN4B, DMKN and CCDC151 exhibited a prominent negative correlation with T cell infiltration. ATP4B exhibited a prominent negative correlation with Chemokine receptors (CCR).

**Fig. 9:**
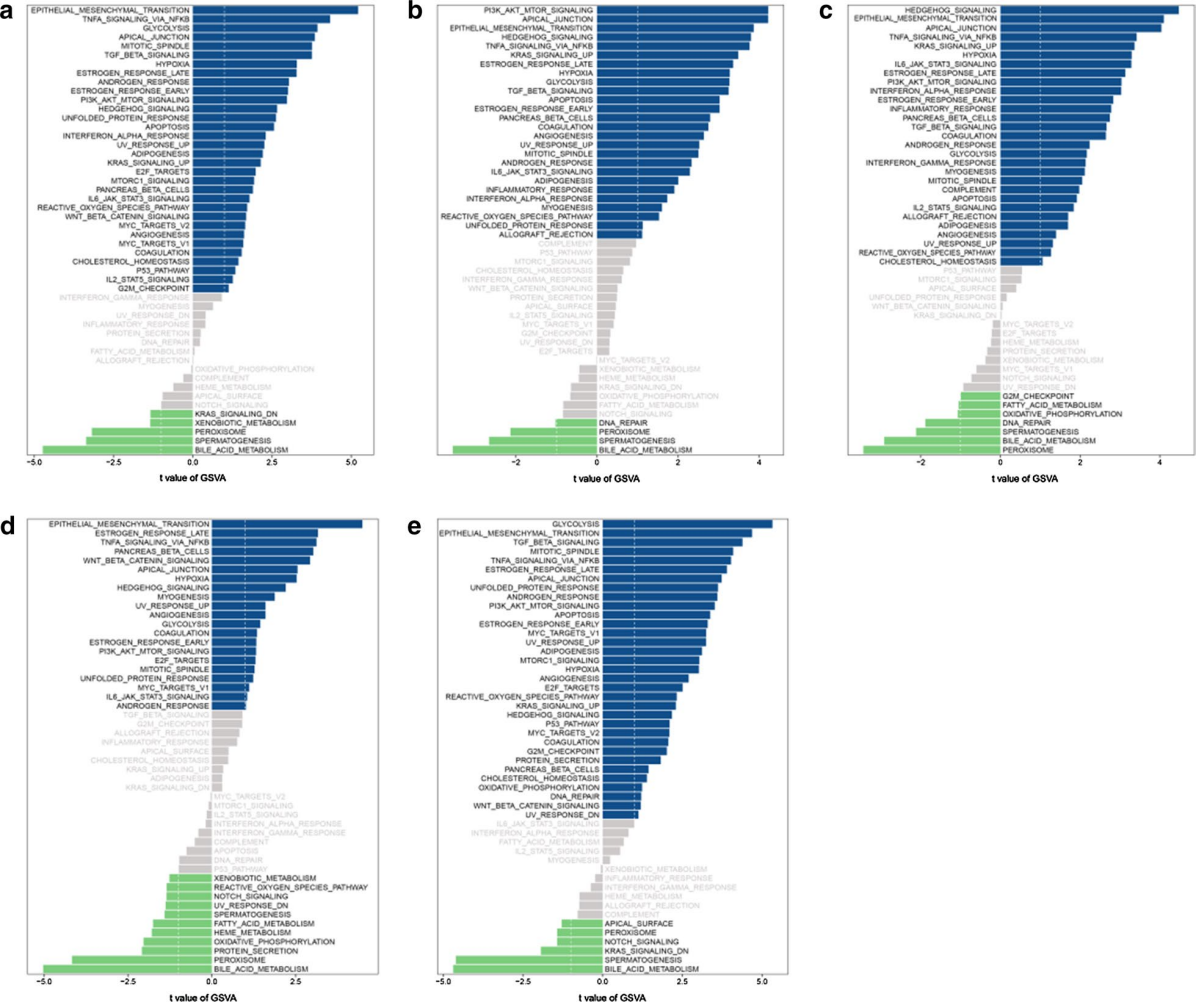
GSVA analysis. **a** ATP4B-enriched pathways. **b** CCDC151-enriched pathways. **c** DMKN-enriched pathways. **d** SCN4B-enriched pathways. **e** TUBA4B-enriched pathways. The blue band represents a positive correlation and the green band represents a negative correlation

Next, we used GSVA to determine the pathways enriched in these five genes. Among the abundant pathways involved in the five genes, we found several overlapping and significantly different pathways related to tumorigenesis, development, and prognosis: PI3K-Akt-mTOR, TNFα-NFKB, and hypoxia (Fig. 9a–e).

## Discussion

The Consortium to Inform Molecular and Practical Approaches to CNS Tumor Taxonomy (cIMPACT-NOW) recently recognized the classification of EPN-PF into two subtypes, according to their methylation levels [22]. The occurrence of tumors is often accompanied by an imbalance in DNA methylation and inactivation of tumor suppressor genes by abnormal methylation is particularly important [23]. Since EPN-PFA is more likely to occur in children under 5 years of age and has a poor prognosis, most of the studies previously conducted have focused on its molecular biological characteristics, namely the deletion mechanism of H3K27me3.

In the present study, we first identified key differential genes between EPN-PFA and EPN-PFB by performing a combined analysis of transcriptome, methylation, and WGCNA, and then analyzed the relationship between the key differential genes and immune pathways in an attempt to explore the role and influence of immune-related pathways in prognosis. Through a multi-omic analysis using expression profiles and methylation profiles, EPN-PF-related DMGs were identified by merging methylated-related highly expressed genes and methylated-related lowly expressed genes together. GO, KEGG, and PPI analyses showed that these DMGs are closely related to the occurrence and development of tumors and immune response pathways such as extracellular matrix organization, neuroinflammatory response, and NF-kappa B signaling pathway.

We then performed a multi-omic WGCNA to systematically identify modules related to EPN-PF based on expression pattern similarities in the 54 samples. In the blue module, which contained 805 genes, we selected 180 hub genes related to EPN-PF. Comparing these genes and the EPN-PF-related DMGs obtained above, we identified five EPN-PF-related hub DMGs: ATP4B, CCDC151, DMKN, SCN4B, and TUBA4B. All of the five genes were highly expressed in EPN-PFB and were less expressed in EPN-PFA. We verified that ATP4B, CCDC151, DMKN, and SCN4B levels were decreased in EPN-PFA compared with EPN-PFB, which was consistent with the predictions. However, perhaps due to the limitation of sample size, the difference in SCN4B was not statistically significant.

The role of these five genes in EPN-PF has never been investigated. The gastric H^+^, K^+^-ATPase(ATP4) is a dimeric heterodimer composed of two catalytic α-subunits and two regulatory β subunits. The β subunits were coded using ATP4B genes [24]. Many studies have revealed that ATP4B expression is decreased in human gastric cancer (GC) [25–28], and some studies have reported abnormal expression of ATP4B in other diseases such as human hepatocellular carcinoma (HCC) [29], laryngopharyngeal reflux, and laryngeal cancer [30]. However, to date, there are no studies about the relationship between this gene and EPN-PF. Similar to the EPN-PF, the ATP4B expression was decreased in GC associated with DNA hypermethylation [31]. A study reported that ATP4B could affect part of nerve function through the immune pathway, and then affect the mental state [32], prompting that ATP4B may affect the prognosis of patients by affecting mental health and immune function.

The CCDC151 gene encodes a coiled-coil protein critical for ODA-complex assembly. Several studies have shown that CCDC151 nonsense mutations can cause primary ciliary dyskinesia (PCD), a complex disease caused by structural or developmental defects that hinder the normal movement of cilia [33–35]. In many organs and systems of humans and mice, movable cilia play a key role in the flow of physiological fluids along the epithelial surface, and in the nervous system, cilia are essential for the normal flow of cerebrospinal fluid (CSF) into the central canal of the spinal cord [36, 37]. A recent study showed that functional loss of CCDC151 could lead to hydrocephalus in a mouse model of primary ciliary dyskinesia [38]. Therefore, the low expression of CCDC151 may affect the prognosis of EPN-PFA children by casing hydrocephalus.

The DMKN gene is located on human chromosome 19q13.12 and encodes 10 putative dermokine (DMKN) transcriptional subtypes in normal epidermis [39]. Studies have confirmed that the abnormal expression of DMKN is related to skin cancer [40], pancreatic cancer [41], colorectal cancer [42], and other cancers, but no study has shown its role in nervous system tumors. However, a report showing that DMKN can affect the activation of STAT3 and down-stream molecular proteins of the MAPK and PI3K signaling pathways suggests that DMKN may play a role in nervous system tumors through this pathway [41].

Sodium voltage-gated channel beta subunit 4 (SCN4B), one of the beta subunits of the sodium channel, regulates the channel gated dynamics and causes voltage-dependent negative shifts in the activation of some α-sodium channels [43]. The variants in SCN4B have been shown to be associated with ventricular tachycardia [44], Huntington’s disease [45], and can be a new biomarker of aggressive cancers [46]. Moreover, Marin et al. showed that SCN4B has a very high interconnection and participation in febrile seizures, nervous disorders, neuromuscular and neurodegenerative diseases, and neurobehavioral manifestations [47]. Therefore, we can boldly speculate that SCN4B is related to EPN-PF. Similar to SCN4B, the low expression of Tubulin alpha 4b (TUBA4B) has also been shown to be significantly associated with poor prognosis of cancer patients [48].

In a variety of human cancers, there is increasing evidence that immunobiology plays an important role in tumor eradication or promotion. The interaction of immunobiological factors in central nervous system malignant tumors has been widely studied [49–52]. Some studies have also confirmed that immunity in EPN-PF plays a role [53, 54], but the number and depth of research is far from enough. Therefore, we conducted the ssGSEA and GSVA algorithms to explore the possible role of EPN-PF-related hub DMGs in the immune system. Through the ssGSEA analysis, we clearly demonstrated that the EPN-PFA and EPN-PFB have significant differences in immunological characteristics, which is consistent with previous studies [54]. Our GSVA analysis showed three crucial pathways related to these five hub genes: PI3K-Akt-mTOR, TNFα-NFKB, and hypoxia.

In a recent study, Michealraj et al. showed that the hypoxic microenvironment is essential for the propagation and growth of EPN-PFA and is associated with poor prognosis [55]. In our study, all five hub genes were connected with hypoxia and confirmed previous research from some aspects. Other enriched biological pathways, such as the PI3K-Akt signaling pathway and extracellular matrix organization, were also closely related to tumor progression and hypoxic microenvironment formation. Moreover, the hypoxic microenvironment could affect the metabolism of tumor cell, induce adaptive changes in cell metabolism, and regulate complex cell signaling pathways, such as NFKB, which participates in inflammatory response and regulates cell proliferation and survival [56, 57].

## Conclusion

In conclusion, we performed a combined analysis of transcriptome, methylation, and WGCNA and identified five novel hypermethylated genes, that were less expressed in EPN-PFA as EPN-PF-related hub genes (ATP4B, CCDC151, DMKN, SCN4B, and TUBA4B), and three of them were confirmed by IHC. Further analysis revealed that these five hub genes could lead to poor prognosis by inducing hypoxia, PI3K-Akt-mTOR, and TNFα-NFKB pathways. Further study of these dysmethylated hub genes in EPN-PF and the pathways they participate in may provides new ideas for EPN-PF treatment.

The findings of this study must be seen in light of some limitations. First, the antibody for TUBA4B was not found; therefore, we did not verify the reliability of its expression difference. Second, due to the limitation of the sample size, we could not verify the differences in SCN4B. Lastly, as our tumor tissues were limited, and some of them were made into paraffin sections, IHC was performed instead of RT-qPCR to verify the expression difference. And we only verify the difference of expression level between the two groups, didn’t detect the methylation level of these genes. In the follow-up experiment, we will collect fresh tumor samples for testing the expression and methylation level difference to compensate for the limitations of this trial.

## Supplementary Information

**Additional file 1: Table S1. The list of DEGs between the two EPN-PF subtypes**

**Additional file 2: Table S2.The list of DMGs between the two EPN-PF subtypes**

**Additional file 3: Table S3. The list of methylated-related highly expressed genes**

**Additional file 4: Table S4. The list of methylated-related lowly expressed genes**

**Additional file 5: Table S5. The list of  180 hub genes in the blue module**

**Additional file 6: Table S6. The gene list and pathways related to each of the 5 hub genes**

## Data Availability

Not applicable.

## Abbreviations

EPN-PF: Posterior fossa ependymoma
WGCNA: Weighted gene co-expression network analysis
GEO: Gene Expression Omnibus
DEGs: Differentially expressed genes
DMGs: Differential methylation genes
EPN: Ependymoma
KEGG: Kyoto Genome Encyclopedia pathway analysis
PPI: Protein–protein interaction
GO: Gene Ontology
WHO: World Health Organization
DAB: 3,3′-Diaminobenzidine
PBS: Phosphate buffer saline
